# Not My Job, I Do Not Want to Do It: The Effect of Illegitimate Tasks on Work Disengagement

**DOI:** 10.3389/fpsyg.2022.719856

**Published:** 2022-04-12

**Authors:** Shuwei Zong, Yi Han, Min Li

**Affiliations:** School of Business Administration, Zhongnan University of Economics and Law, Wuhan, China

**Keywords:** illegitimate tasks, work disengagement, ego depletion, coworker emotional support, leisure crafting

## Abstract

As a prevalent source of work stress, illegitimate tasks (IT) offend employees’ professional identity and threaten individual self-view, then create many negative organizational outcomes. However, current studies have paid inadequate attention to the impact of IT on work disengagement (WD) and its influencing path, failing to comprehensively identify the negative effects of illegitimate tasks. Based on stress-as-offense-to-self (SOS) theory and ego depletion (ED) theory, the influencing path of illegitimate tasks on WD is explored, and coworker emotional support (CES) and leisure crafting (LC) are introduced to explore the intervention conditions on the impact of illegitimate tasks. By analyzing data from a survey of 260 employees, this study reveals the following findings: illegitimate tasks have a significantly positive impact on work disengagement; ED fully transmits the positive impact of illegitimate tasks on work disengagement; CES and LC not only attenuate the effect of illegitimate tasks on ego depletion, but also negatively moderate the indirect effect of illegitimate tasks on work disengagement through ego depletion.

## Introduction

Identifying and helping employees to resolve workplace stress is a topic that has been of great interest to academics and managers. Since Semmer et al. (2005, 2007) proposed the stress-as-offense-to-self (SOS) theory and suggested that individuals perceived events that threaten their positive self-view as stressors, it has been found that illegitimate tasks (IT), a common stressor in organizations, have long been overlooked. IT refer to job duties that employees in a particular job role should not be expected to undertake (Semmer et al., 2010, 2015). Indeed, illegitimate tasks are common in the workplace and individuals develop a perception of illegitimate tasks when the tasks are perceived as unreasonable and unnecessary (Omansky, et al., 2016; Thun, et al., 2018). Therefore, illegitimate tasks include two dimensions: unreasonable tasks and unnecessary tasks (Semmer et al., 2010). Unreasonable tasks are those that the individual believes should be done by others (Omansky, et al., 2016), such as asking a chef who is good at cooking to clean up the dishes in a restaurant. Unnecessary tasks are those tasks that individuals believe should not be done at all and are a waste of their time (Omansky, et al., 2016), such as letting employees record everything carefully, even though the information is simply worthless. The existence of illegitimate tasks deviates from the scientific principle of job design and can cause employees to perceive the nature of tasks, such as “these tasks are not what I am supposed to do” (Semmer et al., 2010), thus arousing employees’ resentment toward the relevant tasks and other negative organizational results (Semmer et al., 2015; Zhou et al., 2018; Ma and Peng, 2019; Schulte-Braucks et al., 2019). Therefore, it is an important issue for scholars and managers to find out the influence path and the intervention conditions of illegitimate tasks (Semmer et al., 2015).

Scholars have conducted extensive research around the consequence of illegitimate tasks (Van Schie et al., 2014; Eatough et al., 2016; Sonnentag and Lischetzke, 2018; Fila et al., 2020). A review of the studies found that illegitimate tasks triggered explicit negative employee behaviors, such as more counter-productive work behaviors, more workplace deviant behaviors, and fewer innovative behaviors (Zhou et al., 2018; Schulte-Braucks et al., 2019). However, the effects of illegitimate tasks on negative working status are less well understood by scholars, and only a few scholars have explored this topic (Semmer et al., 2015; Munir et al., 2017). Compared with explicit negative behaviors, negative working statuses are more harmful and far-reaching because they are difficult to be detected and controlled. Work disengagement (WD) is such a kind of negative working status and is originally conceptualized in relation to work engagement (Kahn, 1990), indicating that employees disengage themselves from their work role and show a mismatch or incompatibility between their ego and their job role (Afrahi et al., 2021). According to SOS theory, illegitimate tasks goes against employees’ perceptions and expectations of their job role (Semmer et al., 2010, 2015), which can lower employees’ self-esteem and evoke negative emotions, inducing resistance to illegitimate tasks and causing employees to exhibit physical, cognitive and emotional withdrawal and self-defense during work role-playing. Hence, WD provides a new perspective for the academic to understand the potential harm of illegitimate tasks. Negative work states, such as work disengagement, can be seen as a signal for employees to express their dissatisfaction (Folkman and Moskowitz, 2004), and if organizations do not detect and intervene it in time, work disengagement of employees is likely to evolve into other deviant behaviors. This suggests that more attention should be paid to the impact of illegitimate tasks on the negative work state of the individual. Unfortunately, there is insufficient discussion in the current literature on the relationship between illegitimate tasks and work disengagement (i.e., whether illegitimate tasks affect work disengagement). Some scholars have explored the relationship between illegitimate tasks and job burnout by including work disengagement as a measurement sub-dimension of job burnout (Demerouti et al., 2001; Semmer et al., 2015), but few studies have examined work disengagement separately. Therefore, this study will explore the relationship between illegitimate tasks and work disengagement and deepen the understanding of the impact of illegitimate tasks on employees’ negative work status.

Besides, extant research has not delved into the mechanisms underlying the impact of illegitimate tasks on work disengagement (i.e., how illegitimate tasks affect work disengagement). Most studies have used justice theory or role theory (Semmer et al., 2015) as research framework and emotional changes or cognitive changes as the mediating explanatory mechanisms (Munir et al., 2017), without conducting research based on the integration mechanism at the level of personal psychological resources. This does not allow academics to fully grasp the impact of illegitimate tasks on work disengagement. In the light of this, SOS theory (Semmer et al., 2007) and ego depletion (ED) theory (Baumeister et al., 1998) are integrated to examine the direct and indirect effects of illegitimate tasks on work disengagement. To be specific, this study introduces the mediating variable ED, which refers to the depletion of self-control after a person has experienced an activity that requires self-control resources (Hagger et al., 2010). Based on this new perspective, this study can explain the proximal negative effects of illegitimate tasks as changes in individual psychological resources, and to further explain the relationship between illegitimate tasks and work disengagement. In other words, confronted with the stress generated by illegitimate tasks, individuals engage in deep cognitive processing and experience more negative emotional experiences, which involves a significant unconscious invocation of self-control resources (Schmidt and Neubach, 2007; Rivkin et al., 2015). When employees deplete their self-control resources excessively, individuals may go into a state of ego depletion soon. In turn, ego depletion leads to individuals being unable to focus on work behaviors, driving them to gradually disengage emotionally, cognitively, and physically from their work role.

Furthermore, scholars have not examined the boundary conditions of illegitimate tasks comprehensively (i.e., when illegitimate tasks affect work disengagement). To date, studies have looked at how factors, such as individual traits (Eatough et al., 2016), power distance orientation (Ahmed et al., 2018), health status (Kottwitz et al., 2013), and leadership style (Apostel et al., 2018), weaken the negative effects of illegitimate tasks but neglected to the examination of external support factors and non-work factors. This is not conducive to improving strategies for resolving illegitimate tasks in management practice. Aiming to solve the above research gap, this study first examines the intervention of coworker emotional support (CES) on illegitimate tasks–ego depletion relationship in terms of external support. CES refers to the extent to which individuals can attach to, rely on, trust, and receive companionship from coworkers when they need help and support in a work situation (Heaney et al., 1993; Liao et al., 2004; Methot et al., 2016). Established research has found that giving appropriate support to persons in stressful work situations can attenuate the negative effects of negative situations (De Clercq et al., 2020), such as supervisor support and coworker support. However, positive supportive behaviors from supervisors can confound employees’ perceptions, which in turn can exacerbate the level of ego depletion for supervisors are the main contributor of employees’ perception of illegitimate tasks (Thau and Mitchell, 2010). And the arrangement and distribution of tasks have little to do with colleagues. Therefore, coworker emotional support is more likely to mitigate the effect of illegitimate tasks on ego depletion. With the emotional support of colleagues, employees have access to valuable external resources that allow them to reduce their cognitive processing load, maintain their self-view, inhibit the generation of negative emotions and alleviate the depletion of their self-control resources in a given situation so that employees do not readily enter a state of ego depletion. Moreover, this study examines the intervention of leisure crafting (LC) on the illegitimate tasks–ego depletion relationship in terms of self-compensation. LC is a kind of proactive behavior taken by individuals during non-work time driven by their own intrinsic motivation (Petrou and Bakker, 2016). Some studies have pointed out that persons do not just react mechanically to the external environment, but implement behaviors that help them reshape the environment (Grant and Ashford, 2008; Parker et al., 2010), and thus provide a buffer for the effects they suffer. This kind of proactive behavior exists in two main domains. One is the work domain, such as job crafting (Wrzesniewski et al., 2010), and the other is the non-work domain, such as leisure crafting (Petrou and Bakker, 2016). However, the ability of employees to craft their work is limited by the availability of opportunities for individual job crafting (Wrzesniewski and Dutton, 2001), and employee autonomy in work situations is very often restricted in many ways. Therefore, this study attempts to explore the moderating effect of leisure crafting on the relationship between illegitimate tasks and ego depletion, starting from the non-work domain where employees have the more autonomous choice. The study expected that leisure crafting would help individuals to replenish their self-control resources, reshape their positive self-view, satisfy their need for autonomy, and awaken their positive emotions, thereby attenuating the negative effects of illegitimate tasks.

Our research has the following three contributions. First, this investigation finds a new perspective (ego depletion) to dissect the impact of illegitimate tasks on work disengagement and to explore the changes in individual psychological resources during this process. Second, the scope of application of SOS theory as well as ego depletion theory is extended. Using both theories together, the direct effect of illegitimate tasks on work disengagement and the role of ego depletion in its transmission process are explained. Finally, the exploration of boundary conditions of illegitimate tasks is facilitated by the introduction of coworker emotional support and leisure crafting.

## Theory and Hypotheses

### Illegitimate Tasks and Work Disengagement

The “illegitimate” nature of the task is closely related to the extent to which the individual perceives the task to be out of his or her role definition (Semmer et al., 2005, 2007). According to SOS theory, illegitimate tasks offend persons’ professional identity and threaten their self-view, thus becoming a source of work stress for persons. Not tied to a given content or a particular form of tasks, illegitimate tasks are subjective “illegitimate” based on a specific job role situation (Semmer et al., 2010, 2015). In general, illegitimate tasks are divided into two dimensions: unreasonable tasks and unnecessary tasks (Semmer et al., 2010). Unreasonable tasks refer to tasks that are not considered to be part of an individual’s professional role (Omansky, et al., 2016), which reflects the misalignment of task assignment targets in the eyes of persons. There are four types of unreasonable tasks: (a) tasks that are outside the scope of the individual’s profession; (b) tasks that are incompatible with the individual’s professional status; (c) tasks that do not reflect one’s professional identity but are demanding; and (d) tasks that put the individual in an awkward situation. Unnecessary tasks refer to tasks that employees assume no one should be performing (Omansky, et al., 2016), which shows individuals’ fundamental denial of the value and meaning of such tasks. Unnecessary tasks consist of three cases: (a) tasks that make no sense at all; (b) tasks that could be avoided or completed in less time if they were properly scheduled; and (c) tasks that are to satisfy the leader’s preferences simply.

Stress-as-offense-to-self theory emphasizes that individuals have a need to maintain a positive self-view and that their self-view can be threatened in stressful situations (Semmer et al., 2007). Illegitimate tasks go against employees’ perceptions and expectations of the job role (Semmer et al., 2010, 2015). This makes being assigned illegitimate tasks prone to the individual’s perception of professional role identity violation and creates a self-offensive stressor in the work context. And the self-view threat created by illegitimate tasks can trigger work disengagement by lowering self-esteem and generating negative emotions in employees. Specifically, for one thing, individuals want to strive to maintain a positive self-view (Sedikides and Strube, 1997) and consciously or unconsciously examine their self-view through attention to external information. Task assignment sends specific social messages and signals, which affects one’s measure of self-view. Research has found that when an individual recognizes that he or she does not exist as a professional, his/her sense of self-worth and self-meaning will significantly reduce (Schulte-Braucks et al., 2019). This makes people who are assigned illegitimate tasks not only aggravate their cognitive burden through task assignment information, but also perceive the work itself as a potential threat to their self-view and thus prone to a state of low self-esteem (Sonnentag and Lischetzke, 2018; Schulte-Braucks et al., 2019). In response to this offense and threat, individuals opt to reduce their resource investment in work tasks and exhibit certain defensive behaviors or self-distancing. That is, illegitimate tasks lead the individual to a state of work disengagement.

For another thing, according to SOS theory, the perception and experience of social devaluation may influence individual negative discrete emotions (Eatough et al., 2016). It has been found that individuals who perceive illegitimate tasks develop negative emotional reactions, such as resentment (Semmer et al., 2015), anger (Zhou et al., 2018), anxiety, and depression (Fila and Eatough, 2020). In modern organizations, employees are not only task bearers but also relatively repressed objects. This makes individuals’ negative perception of illegitimate tasks form persistent pressure, take up employees’ cognitive resources, and induce negative emotions of individuals. Furthermore, employees will experience cognitive disengagement and emotional disengagement and reduce the degree to which individuals relate to their work roles and engage in their work, ultimately giving rise to work disengagement. Based on the previous discussion, the following hypothesis was proposed:

*Hypothesis 1*: Illegitimate tasks have a positive impact on work disengagement.

### Illegitimate Tasks, Ego Depletion, and Work Disengagement

Ego depletion theory suggests that individuals participate in volitional activities, including cognitive processing, making choices, behavioral control, and active response all result in depletion of self-control resources (Baumeister et al., 1998). Faced with a stressor created by an illegitimate task, the individual makes a lot of unconscious invocation on his/her resources (Schmidt and Neubach, 2007; Rivkin et al., 2015), especially self-control resources. When the employee’s reserves of self-control resources are insufficient, he/she will fall into ego depletion. More precisely, illegitimate tasks can affect ego depletion in both cognition and emotion ways.

From a cognitive perspective, when assigned an illegitimate task that does not match their professional role, employees develop deeper processing about the reasons for this assignment, which in turn increases the cognitive burden on the individual and depletes his/her psychological resources (Thau and Mitchell, 2010). Subsequently, employees question their ability to assume their core professional role in the future and thus become more cognitively concerned with the exception task they receive to determine whether it is reasonable and necessary. This persistent concern implies that illegitimate tasks have a progressive negative impact on persons’ cognitive and attentional load, which may entail a deepening level of resource depletion. What is more, negative subjective perceptions of oneself can bring about depletion of resources. Illegitimate tasks undermine the individual’s self-concept (Semmer et al., 2010, 2015), which triggers the perception of unfairness (Ahmed et al., 2018), and low organizational status (Cropanzano et al., 2001). As a consequence, being assigned to perform illegitimate tasks will lead to a higher degree of ego depletion. From an emotional perspective, persons who are in a negative mood will have higher levels of ego depletion (Shmueli and Prochaska, 2012). Being assigned illegitimate tasks means that individuals need to take on tasks that should not be theirs, which leads to the perception of effort–reward imbalance (Omansky et al., 2016), as well as sadness and depression that their professional value is not recognized or accepted (Eatough et al., 2016). Then, individuals need to consume certain self-control resources to process these emotions in order to diminish the timely negative effects that such negative emotions can have on them, which further exacerbates the degree of ego depletion.

Meanwhile, ego depletion theory states that individual resources are limited. When the self-control resources are depleted in the earlier activity, it will be difficult for people to perform self-control and volitional regulation for other possible subsequent behaviors (Baumeister et al., 2007). Therefore, this research believed that when individuals consume their self-control resources in illegitimate tasks, they will not be able to focus on engaging in work behaviors either consciously or unconsciously, which will bring about the emergence of work disengagement. Furthermore, persons in a state of ego depletion are more sensitive to timely rewards and are more likely to focus on short-term benefits (Faralla et al., 2017). This suggests that employees will keep a watchful eye on non-work behaviors that provide them with timely positive emotions or benefits than work behaviors that are characterized by “immediate input-delayed reward.” It has been found that individuals who are in ego depletion produce more behaviors against the rules and regulations (Yam et al., 2014), less proactive behavior, and employees have lower task adaptability (Deng et al., 2016). In other words, ego depletion causes the attention and energy that individuals can focus on their work to be distracted or diverted, leading them to gradually detach emotionally, cognitively, and physiologically from their work roles. Thus, illegitimate tasks can bring employees into a state of work disengagement through ego depletion. Based on the previous discussion, the following hypothesis was proposed:

*Hypothesis 2*: Ego depletion mediates the relationship between illegitimate tasks and work disengagement.

### The Moderating Effect of Coworker Emotional Support

Coworker emotional support can provide people with the external resources they need, allowing them to relax mentally and alleviating the depletion of their self-control resources in a given situation (Sloan, 2012; Methot et al., 2016). Based on SOS theory and ego depletion theory, coworker emotional support help individuals avoid or diminish the stressful damage of illegitimate tasks in three ways: cognitive processing burden reduction, self-view maintenance, and negative emotion relief.

To begin with, coworkers can use their own experiences to help the employee interpret the situation he/she is encountering and provide additional information (Methot et al., 2016), enabling the employee to view this task assignment from a new perspective and avoiding him/her from falling into cognitive anxiety about “why was I assigned an illegitimate task?,” thus lessening the depletion of self-control resources through the reduction of cognitive processing burden. Next, a significant reason why illegitimate tasks can be stressfully harmful to people is that employees want to work hard to maintain a positive self-view, while illegitimate task assignments send the message that they are not respected, valued, or appreciated (Eatough et al., 2016; Kottwitz et al., 2019). This makes individuals fear that the image of self in the eyes of others will be harmed. Coworker emotional support conveys signals of concern, respect, and trust (Chou and Robert, 2008). This perception of positive interpersonal interactions leads individuals to believe that they are in a friendly and healthy interpersonal relationship and satisfy their interpersonal needs. In line with SOS theory (Semmer et al., 2007), when a positive image of the individual is maintained, the damage caused by stress to the individual is diminished. Finally, in a high coworker emotional support situation, persons are able to communicate effectively and harmoniously with their coworkers and receive their concerns (Zhou and George, 2001). Communication filled with goodwill can infuse positive emotions into individuals and dissolve negative emotions, such as anger and depression, brought about by having to perform illegitimate tasks, thus weakening ego depletion caused by illegitimate tasks. Based on the previous discussion, the following hypothesis was proposed:

*Hypothesis 3*: Coworker emotional support negatively moderates the relationship between illegitimate tasks and ego depletion.

### The Moderating Effect of Leisure Crafting

Leisure crafting is an individual’s initiative to engage in leisure activities based on goals, such as interpersonal relationship building, learning, and personal growth (Petrou and Bakker, 2016), and is effective in increasing self-control resources, which in turn positively influences the individual’s subsequent level of work engagement (Ragsdale and Beehr, 2016). Also, research has found that leisure activities can act as a compensatory mechanism when an individual’s needs or will are frustrated (Berg et al., 2010; Vogel et al., 2016). Thus, while individuals experience a lack of self-control resources and a threat to their self-view caused by illegitimate tasks, leisure crafting can attenuate the negative effects of illegitimate tasks and decrease the level of ego depletion through replenishing self-control resources, reshaping a positive self-view, satisfying autonomy needs and arousing positive emotions.

At first, leisure crafting allows individuals to undergo the challenge of strengthening their sense of self-control to learn new things or develop themselves (Petrou and Bakker, 2016). This provides individuals with a supplement of self-control resources, ensuring that the individual has sufficient resources to cope with illegitimate tasks. According to ego depletion theory (Baumeister et al., 1998), individuals are able to control and manage subsequent behavior when self-control resources are sufficient. Meanwhile, leisure crafting enables employees to continue to develop themselves in non-work areas and to continuously improve their sense of self-efficacy (Bandura, 1993). This satisfies the individual’s need to maintain and realize self-worth, thereby alleviating the low self-worth perceptions associated with engaging in illegitimate tasks. Then, leisure crafting can attenuate the perception of self-view threat caused by illegitimate tasks *via* reshaping the individual’s positive self-view. An important reason why illegitimate tasks become a source of stress for employees is that individuals want to strive to maintain a positive self-view. When employees engage in proactive leisure activities with colleagues and others during non-working hours, they may regain positive affirmation from others (Snir and Harpaz, 2002). This creates an opportunity for employees to reshape a positive self-view, thus reducing the threat to self-view posed by illegitimate tasks.

In addition, leisure crafting offers a great deal of autonomy to employees, satisfying individual needs for autonomy (Deci and Ryan, 2000) and reducing the loss of self-control resources due to illegitimate tasks. The lack of autonomy from illegitimate tasks, which are not tasks that employees choose and accept freely, aggravates employees’ sense of restraint at work, which in turn leads to negative emotions and perceptions in the work situation. Leisure crafting, in contrast, provides employees with the ability and opportunity to regain control and self-determination during non-working hours, which can mitigate the negative impact of illegitimate tasks. Finally, leisure crafting can evoke positive emotions and help employees better deal with the negative emotional experiences associated with illegitimate tasks, consequently buffering the negative effects of illegitimate tasks. Research has found that individuals who engage in leisure activities have a greater positive affect and life satisfaction (Wiese et al., 2018), develop less depression (Sonnentag, 2001), and increase next-day vitality by reinforcing psychological detachment and relaxation during the non-work time (Ten Brummelhuis and Bakker, 2012). This suggests that leisure crafting has positive spillover effects on the work domain (Hecht and Boies, 2009), creating positive emotional states for individuals, alleviating negative emotions (Boon, 2006; Greenhaus and Powell, 2006) from illegitimate tasks, and reducing ego depletion. Based on the previous discussion, the following hypothesis was proposed:

*Hypothesis 4*: Leisure crafting negatively moderates the relationship between illegitimate tasks and ego depletion.

### Moderated Mediation Effect

Research has shown that high coworker emotional support can attenuate the negative effects of work stressors (Mathieu et al., 2019). In other words, with high coworker emotional support, the depletion of self-control resources from illegitimate tasks is limited, which does not significantly increase work disengagement. According to ego depletion theory (Baumeister et al., 1998), the usage and consumption of resources exacerbate the negative effects of ego depletion, while the supplementation of new resources relieves the individual’s negative emotions and thus diminishes the effects of ego depletion. Similarly, persons with high leisure crafting were able to obtain complementary need satisfaction and show stronger positive emotional disposition during non-work time, enabling them to better handle and cope with resource depletion-induced work disengagement. Based on the previous discussion, the following hypotheses were proposed:

*Hypothesis 5*: Coworker emotional support negatively moderates the mediation effect of ego depletion on the relationship between illegitimate tasks and work disengagement.

*Hypothesis 6*: Leisure crafting negatively moderates the mediation effect of ego depletion on the relationship between illegitimate tasks and work disengagement.

**Figure 1:**
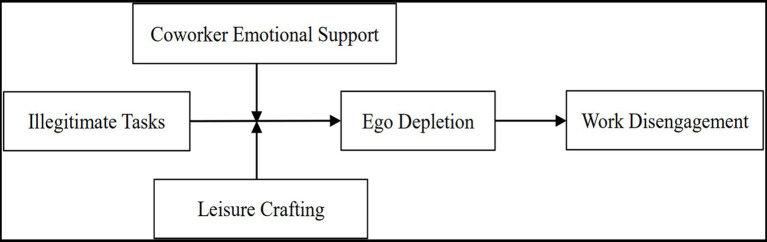
Research conceptual model.

Based on the above hypothesis analysis, the research conceptual model for this study is shown in Figure 1.

## Materials and Methods

### Data Collection

Full-time employees from four Chinese companies were contacted using the convenience sampling technique in this research. These companies were from a variety of industries, such as foreign trade and real estate. To address the issue of common method bias, this study conducted data collection at two rounds, separated by approximately half a month. In the first round, employees were asked to fill in information about his/her demographic variables, as well as illegitimate tasks, leisure crafting, and coworker emotional support scales. In the second round, employees self-reported their information on ego depletion and work disengagement.

A total of 350 employees were invited and 310 were actually collected, with a response rate of 88.6%. To ensure data quality, three ways of filtering were applied to the data: (1) delete samples with too many missing values; (2) delete samples of all items filled with the same answers; and (3) delete samples of the same answers in both reverse and forward items. Eventually, 50 invalid responses were eliminated, and 260 valid responses were gained, with a valid response rate of 74.3%. Among these respondents, 45.4% were male and 54.6% were female, indicating an even distribution of male and female proportions. According to age composition, the respondents were mainly aged 31–40 years old (accounting for over 50%), followed by 18–30 years old (accounting for 41.5%), which was consistent with the current organizational situation that the labor force is mainly young and middle-aged. In terms of education level, 70% had a bachelor’s degree (23.1% had a college degree and below, 6.9% have a master’s degree and above), which was in line with the status quo of employees in most organizations. The average organizational tenure (OT) was 2.108 years (SD = 0.722).

### Measurement

Established scales from relevant foreign studies were used to measure the variables in this study. To ensure the accuracy and comprehensibility of the scales, a standard “translation and back-translation” procedure was strictly followed for the foreign scales to make them fit the Chinese cultural context (Brislin, 1980). Each scale was completed by the employees and scored using a five-point Likert scale. It should be noted that scores 1–5 on Illegitimate Tasks Scale and Ego Depletion Scale represent “never,” “occasionally,” “sometimes,” “often,” and “always,” respectively; scores 1–5 on Work Disengagement Scale, Coworker Emotional Support Scale, and Leisure Crafting Scale represent “strongly disagree,” “basically disagree,” “neither agree nor disagree,” “basically agree,” and “strongly agree,” respectively.

Illegitimate tasks were measured using the scale revised by Semmer et al. (2010). The scale contains eight items, four of which measure the unreasonable tasks, such as “You need to handle tasks that should be done by someone else,” and four of which measure the unnecessary tasks, such as “You need to handle tasks that are completely unnecessary.” The reliability coefficient for the scores for this sample was 0.83.

Work disengagement was measured using the scale developed by Demerouti et al. (2001). The scale contained eight items, three of which were positive items, such as “I feel that I will gradually become detached from my work in the long run,” and five of which were negative items, such as “I feel that I am becoming more and more attached to my work.” The reliability coefficient for the scores for this sample was 0.78.

Ego depletion was measured using the scale revised by Johnson et al. (2014). The scale contains five items, with specific questions, such as “I feel unable to concentrate my thoughts.” The reliability coefficient for the scores for this sample was 0.78.

Coworker emotional support was measured using the scale developed by Methot et al. (2016). The scale contains five items, such as “My coworker share relevant personal experiences to provide an alternative perspective to solve my problems.” The reliability coefficient for the scores for this sample was 0.78.

Leisure crafting was measured using the scale developed by Petrou and Bakker (2016). The scale contains nine items, such as “I often have novel experiences through active leisure activities.” The reliability coefficient for the scores for this sample was 0.81.

The demographic variables have been found to have a possible impact on ego depletion and work disengagement (Courtright et al., 2016; Aslam et al., 2018). Therefore, this study controlled for relevant demographic variables, including gender, age, education, and OT.

## Data Analysis

### Reliability and Validity Test

From the above analysis, it can be seen that Cronbach’s α coefficient of each scale is above 0.7, indicating the high reliability of each scale. In this study, Mplus 8.3 software was used to compare the multi-factor models and then determine the best model. Also, for purpose of reducing the number of entries in each factor to a reasonable level, the packing method was employed in this paper (Little et al., 2013). Table 1 showed the result of confirmatory factor analysis. The five-factor model, four-factor model, three-factor model, two-factor model, and one-factor model were compared and analyzed, and the results showed that the five-factor model fit better (*χ*^2^/df = 1.392, RMSEA = 0.039, CFI = 0.976, and TLI = 0.968), proving that the five variables had better discriminant validity among them.

**Table 1:** Results for confirmatory factor analyses.

Model	Factor	*χ* ^2^	df	*χ*^2^/df	RMSEA	CFI	TLI
Five-factor model	IT;ED;WD;CES;LC	111.366	80	1.392	0.039	0.976	0.968
Four-factor model	IT;ED;WD;CES + LC	233.172	84	2.776	0.083	0.885	0.857
Three-factor model	IT + ED;WD;CES + LC	308.696	87	3.548	0.099	0.829	0.794
Two-factor model	IT + ED + WD;CES + LC	470.696	89	5.289	0.128	0.706	0.654
One-factor model	IT + ED + WD + CES + LC	770.277	90	8.559	0.171	0.477	0.389

IT, illegitimate tasks; ED, ego depletion; WD, work disengagement; CES, coworker emotional support; and LC, leisure crafting.

### Descriptive Analysis

The means, SDs, and correlation coefficients of the variables were presented in Table 2. According to Table 2, illegitimate tasks were positively correlated with work disengagement (*r* = 0.385, *p* < 0.01) and ego depletion (*r* = 0.616, *p* < 0.01); ego depletion was positively correlated with work disengagement (*r* = 0.583, *p* < 0.01). Besides, the data for all five variables in this study were obtained from a single source, so common method bias could be a threat to this study (Podsakoff et al., 2003). In this regard, the Harman single factor test was used to test for the presence of common method bias (Podsakoff et al., 2003). If the exploratory factor analysis extracted only one factor or if the variance explanation rate of a factor exceeded 40%, a serious problem of common method bias was indicated. Test results showed that the largest factor had an explanation of variance of 21.816%, indicating that there was no significant common method bias in this study.

**Table 2:** Descriptive statistics and correlations of variables.

Variable	1	2	3	4	5	6	7	8	9
1. Sex	−								
2. Age	−0.103	−							
3. Education	0.066	−0.085	−						
4. OT	−0.153^*^	0.689^**^	−0.041	−					
5. IT	−0.044	−0.092	−0.060	−0.108	−				
6. CES	0.076	−0.091	−0.112	−0.027	−0.010	−			
7. LC	0.029	−0.133^*^	−0.074	−0.148^*^	0.052	0.440^**^	−		
8. ED	−0.098	−0.041	−0.104	−0.058	0.616^**^	−0.273^**^	−0.208^**^	−	
9. WD	0.031	−0.062	0.085	−0.103	0.385^**^	−0.484^**^	−0.452^**^	0.583^**^	−
Mean	1.546	1.665	2.762	2.108	2.867	3.885	3.738	2.318	2.208
SD	0.499	0.639	0.690	0.722	0.632	0.626	0.563	0.704	0.583

* *p* < 0.05;

** *p* < 0.01.

OT, organizational tenure; IT, illegitimate tasks; ED, ego depletion; WD, work disengagement; CES, coworker emotional support; and LC, leisure crafting.

## Results

### Test of Main Effect and Mediating Effect

To start with, this research examined the effect of illegitimate tasks on work disengagement. The results of the analysis were illustrated in Table 3. Table 3 revealed that after controlling for the effects of gender, age, education, and organizational tenure, illegitimate tasks had a significantly positive impact on work disengagement (*β* = 0.358, *p* < 0.001), whereby hypothesis 1 held.

**Table 3:** Results for main effect analysis.

	*β*	SE	*t*	LLCI	ULCI	*R* ^2^	*F*
Outcome variable: work disengagement							
Constant	0.946	0.276	3.426^***^	0.402	1.489	0.165	10.059^***^
Sex	0.038	0.068	0.563	−0.096	0.172		
Age	0.037	0.072	0.507	−0.106	0.179		
Education	0.090	0.049	1.847	−0.006	0.186		
Organizational tenure	−0.064	0.065	−0.990	−0.191	0.063		
Illegitimate tasks	0.358	0.053	6.704^***^	0.253	0.464		

LL, low limit; CI, confidence interval; UL, upper limit. Bootstrap size = 5,000.

*** *p* < 0.001.

Then, this study utilized the PROCESS macro outlined by Hayes (2013) to test the mediating effect of ego depletion in the relationship between illegitimate tasks and work disengagement. The results of the analysis were presented in Table 4. Table 4 demonstrated that illegitimate tasks positively affected ego depletion (*β* = 0.679, *p* < 0.001), and ego depletion still had a significant positive effect on work disengagement (*β* = 0.481, *p* < 0.001) after controlling for the effects of illegitimate tasks. More importantly, the indirect effect estimate for ego depletion between illegitimate tasks and work disengagement was 0.327 with a 95% CI of [0.239, 0.428] and the interval did not contain 0, indicating a significant indirect effect. This indicated that ego depletion played a mediating role between illegitimate tasks and work disengagement. Thus hypothesis 2 was supported.

**Table 4:** Results for mediating effect analysis.

	*β*	SE	T	LLCI	ULCI	*R* ^2^	*F*
Outcome variable: ego depletion							
Constant	0.702	0.285	2.462^*^	0.140	1.263	0.389	32.358^***^
Sex	−0.096	0.070	−1.363	−0.234	0.043		
Age	0.014	0.075	0.182	−0.134	0.161		
Education	−0.064	0.050	−1.269	−0.163	0.035		
Organizational tenure	−0.014	0.067	−0.206	−0.145	0.117		
Illegitimate tasks	0.679	0.055	12.300^***^	0.570	0.788		
Outcome variable: work disengagement							
Constant	0.608	0.243	2.504^*^	0.130	1.086	0.371	24.909^***^
Sex	0.084	0.059	1.421	−0.033	0.201		
Age	0.030	0.063	0.479	−0.094	0.154		
Education	0.121	0.043	2.840^**^	0.037	0.205		
Organizational tenure	−0.057	0.056	−1.021	−0.168	0.053		
Illegitimate tasks	0.032	0.059	0.539	−0.084	0.147		
Ego depletion	0.481	0.053	9.107^***^	0.377	0.585		
Indirect effect of X on Y	0.327	0.048	−	0.239	0.428		

LL, low limit; CI, confidence interval; and UL, upper limit. Bootstrap size = 5,000. X = illegitimate tasks; Y = work disengagement.

* *p* < 0.05;

** *p* < 0.01;

*** *p* < 0.001.

### Test of Moderating Effect

Hierarchical regression analysis was used to examine the moderating effect of coworker emotional support and leisure crafting in the relationship between illegitimate tasks and ego depletion, and the detailed results were presented in Table 5. Model 2 revealed that the interaction coefficient of coworker emotional support and illegitimate tasks was significantly negatively related to ego depletion (*β* = −0.249, *p* < 0.001) after controlling for the effects of demographic variables as well as main effects, indicating that coworker emotional support had a negative moderating effect in the relationship between illegitimate tasks and ego depletion, namely, coworker emotional support could weaken the positive effect of illegitimate tasks on ego depletion. Hypothesis 3 was supported. Model 4 showed that the interaction coefficient of leisure crafting and illegitimate tasks was significantly negatively related to ego depletion (*β* = −0.225, *p* < 0.001), indicating that leisure crafting had a negative moderating effect in the relationship between illegitimate tasks and ego depletion, namely, leisure crafting could alleviate the resource loss of employees from illegitimate tasks, and the degree of ego depletion would be reduced. Hypothesis 4 was supported.

**Table 5:** Results for moderating effect analysis.

Variable	Ego depletion			
	Model 1	Model 2	Model 3	Model 4
Sex	−0.064	−0.055	−0.091	−0.085
Age	−0.033	−0.024	−0.005	0.004
Education	−0.100^*^	−0.107^*^	−0.085^*^	−0.074
Organizational tenure	0.009	−0.006	−0.038	−0.052
Illegitimate tasks	0.673^***^	0.655^***^	0.688^***^	0.704^***^
Coworker emotional support	−0.312^***^	−0.365^***^		
Illegitimate tasks * Coworker emotional support		−0.249^***^		
Leisure crafting			−0.315^***^	−0.329^***^
Illegitimate tasks * leisure crafting				−0.225^**^
*R* ^2^	0.464	0.489	0.450	0.465
Adj.*R*^2^	0.451	0.475	0.437	0.450
F	36.445	34.439	34.541	31.254

* *p* < 0.05;

** *p* < 0.01;

*** *p* < 0.001.

**Figure 2:**
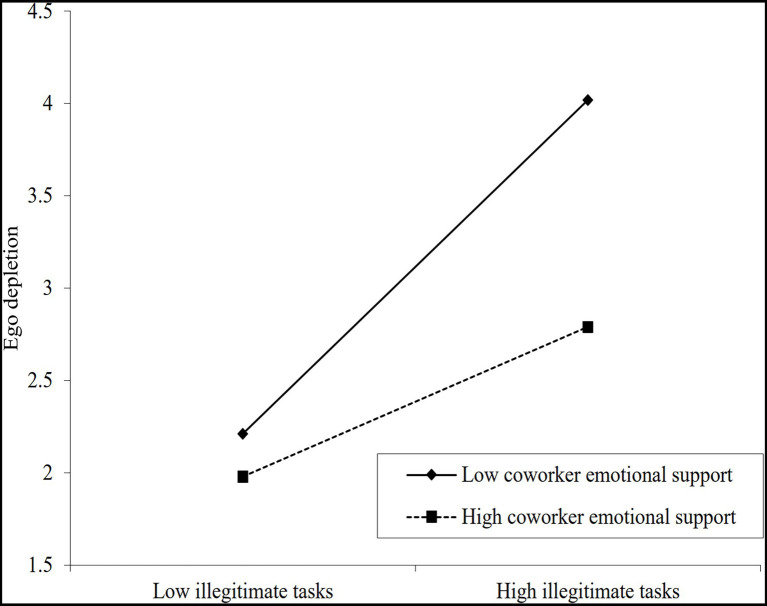
Moderating effect of coworker emotional support on relationship between illegitimate tasks and ego depletion.

**Figure 3:**
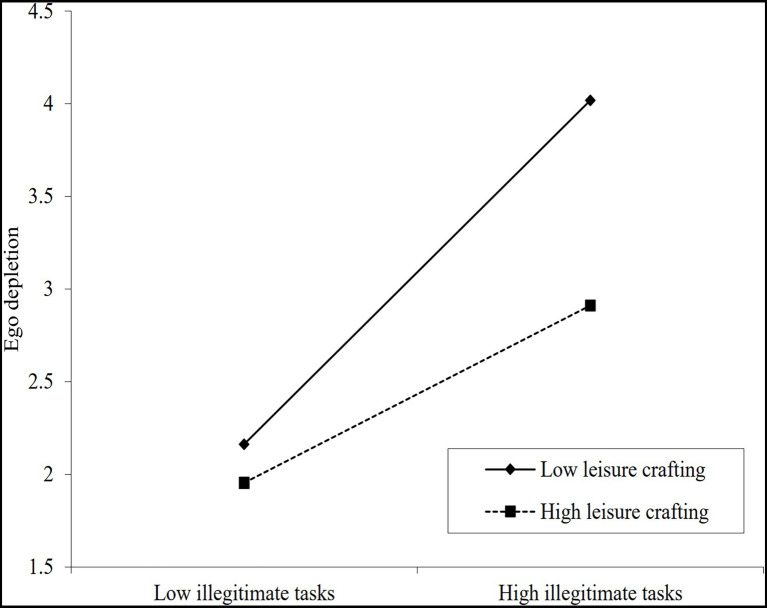
Moderating effect of leisure crafting on relationship between illegitimate tasks and ego depletion.

To further explain the moderating effects, this study conducted the simple slope test and plotted the moderating effects of coworker emotional support as well as leisure crafting on the relationship between illegitimate tasks and ego depletion, as shown in Table 6 and Figures 2, 3. The results showed that the positive effect of illegitimate tasks on ego depletion was weaker under high coworker emotional support compared to low coworker emotional support, and the positive effect of illegitimate tasks on ego depletion was weaker under high leisure crafting compared to low leisure crafting. This further corroborates our hypotheses.

**Table 6:** Results for simple slop tests.

	Effect	SE	T	LLCI	ULCI
M(CES)−1SD	0.811	0.064	12.684^**^	0.685	0.937
M(CES)	0.655	0.051	12.846^**^	0.555	0.755
M(CES)+1SD	0.499	0.071	7.056^**^	0.360	0.638
M(LC)−1SD	0.830	0.075	11.011^**^	0.682	0.979
M(LC)	0.704	0.052	13.462^**^	0.601	0.807
M(LC)+1SD	0.577	0.067	8.585^**^	0.444	0.709

LL, low limit; CI, confidence interval; and UL, upper limit. Bootstrap size = 5,000. CES, coworker emotional support; LC, leisure crafting.

** *p* < 0.01.

### Test of Moderated Mediation Effect

In addition, this research wanted to explore whether indirect effects transmitted by mediating variables differed significantly across levels of moderating variables. Using PROCESS macro, this study calculated the conditional indirect effects of illegitimate tasks on work disengagement (through ego depletion) at three values of coworker emotional support (or leisure crafting). The results of the analysis were shown in Table 7. Firstly, coworker emotional support could negatively moderate the mediating effect of ego depletion in illegitimate tasks–work disengagement relationship. As can be seen from Table 7, the corresponding 95% CI for the high level of coworker emotional support (mean plus 1 SD) was [0.169,0.351], which did not contain 0; the corresponding 95% CI for the low level of coworker emotional support (mean minus 1 SD) was [0.283,0.486], which did not contain 0. Also, the index of moderated mediation (See Table 8) was significant [95% CI: (−0.216, −0.063)], indicating that coworker emotional support significantly moderates the mediating effect of ego depletion. Thus, hypothesis 5 was supported.

**Table 7:** Results for conditional indirect effect analysis.

	Effect	Boot SE	Boot LLCI	Boot ULCI
M(CES)−1SD	0.373	0.051	0.283	0.486
M(CES)	0.301	0.045	0.220	0.398
M(CES)+1SD	0.253	0.047	0.169	0.351
M(LC)−1SD	0.394	0.059	0.283	0.516
M(LC)	0.334	0.046	0.251	0.431
M(LC)+1SD	0.274	0.051	0.179	0.384

Bootstrap size = 5,000, bootstrap CI = 95%. LL, low limit; CI, confidence interval; and UL, upper limit. CES, coworker emotional support; LC, leisure crafting.

**Table 8:** Index of moderated mediation.

	Index	Boot SE	Boot LLCI	Boot ULCI
CES	−0.120	0.038	−0.216	−0.063
LC	−0.108	0.054	−0.220	−0.007

Bootstrap size = 5,000, bootstrap CI = 95%. LL, low limit; CI, confidence interval; and UL, upper limit. CES, coworker emotional support; LC, leisure crafting.

Next, leisure crafting could negatively moderate the mediating effect of ego depletion in illegitimate tasks–work disengagement relationship. Table 7 indicated that under high leisure crafting (mean plus 1 SD), its corresponding 95% CI was [0.179, 0.384], which did not contain 0; under low leisure crafting (mean minus 1 SD), its corresponding 95% CI was [0.283, 0.516], which did not contain 0. Also, the index of moderated mediation (See Table 8) was significant [95% CI: (−0.220, −0.007)], indicating that leisure crafting significantly moderates the mediating role of ego depletion. Thus, hypothesis 6 was supported.

## Discussion and Conclusion

### Discussion

Based on SOS theory and ego depletion theory, this paper explored the influence of illegitimate tasks on work disengagement and verified the boundary of illegitimate tasks in terms of both external support and self-compensation. The main findings of this study were as follows. At first, as a new type of stressor, illegitimate tasks could induce work disengagement in employees. Illegitimate tasks triggered the need for self-control by causing the loss of cognitive and emotional resources and became a stressful situation that depleted the individual’s self-control resources. Continued exposure to such situations would further reduce closeness to the work role and effort to engage in the work, ultimately giving rise to work disengagement. This was consistent with finding of Bennett and Robinson (2000) that the discrepancy between work reality and self-expectations motivated persons to lessen the discrepancy and express their dissatisfaction.

Next, ego depletion fully transmitted the effect of illegitimate tasks on work disengagement. Illegitimate tasks could decrease individuals’ self-control resources by creating cognitive load, triggering negative self-perceptions and negative emotions, which could lead them to a state of ego depletion and make it difficult for them to focus on work engagement, causing them to develop work disengagement. This was in line with existing research (Faralla et al., 2017) which suggested that employees in a state of ego depletion were more sensitive to timely rewards and more likely to focus on short-term benefits.

Besides, coworker emotional support mitigated the negative effects of illegitimate tasks. On the one hand, by satisfying the interpersonal needs and positive emotions, coworker emotional support reduced the threat of self-view and dissolved the negative emotions caused by illegitimate tasks, cutting down the depletion of self-control resources. On the other hand, coworker emotional support also weakened the impact of illegitimate tasks on work disengagement. This extended the moderating effect of coworker emotional support to individual behavior and was consistent with present research findings (Chae et al., 2019) that individual access to resources had a debilitating effect on negative outcomes. Furthermore, it is interesting and worth exploring what role supervisor support, an important part of organizational support (Chae et al., 2019; Mathieu et al., 2019), would play in coping with illegitimate tasks. Subsequent research could compare the differentiated results produced by coworker support and supervisor support as moderating variables.

Finally, leisure crafting eased the negative effects of illegitimate tasks. Leisure crafting, as a non-work positive activity, could exert spillover and compensatory effects by satisfying individual autonomy needs and increasing self-efficacy (Vogel et al., 2016), lessening the depletion of personal internal coordination. Also, leisure crafting dampened the effects of illegitimate tasks on work disengagement. This was because leisure crafting provides people with an opportunity to deal with negative work situations, which extended positive emotions to work situations, and in turn weakened the negative effect of negative situational factors (Vogel et al., 2016).

### Theoretical Implications

Several theoretical contributions existed in this study. First of all, this study expanded the research perspective on the impact of illegitimate tasks on individual outcomes. Most of the ongoing researches on illegitimate tasks were based on a single perspective of cognition or emotion, which could only partially explain the negative effects of illegitimate tasks. In this regard, it was necessary to break the “either/or” perspective of cognition or emotion when exploring the influence of illegitimate tasks. Therefore, this study introduced the mediating role of ego depletion, explored the explanatory mechanism of the relationship between illegitimate tasks and work disengagement from the perspective of individual psychological resources, and revealed the dynamics of psychological resources in the process of illegitimate tasks affecting work disengagement according to ego depletion theory.

Then, a theoretical basis for introducing the illegitimate tasks into the dual-task paradigm experiment was provided. Most of the current studies had been conducted by scholars through the experimental group members’ conscious avoidance of thinking about a concept or thing in their own thinking activities (Tice et al., 2007), ignoring other environmental stimuli and focusing on them (Baumeister et al., 1998), restraining or amplifying their emotional reactions (Hofmann et al., 2007), controlling habitual behavioral tendencies (Gailliot et al., 2007), and resisting food temptations (Shmueli and Prochaska, 2012) trigger the depletion of individual self-control resources. Based on the exploration of the relationship between illegitimate tasks and ego depletion, this study found that illegitimate tasks positively predicted individual resource depletion, which in turn expanded the excitation conditions for ego depletion and provided theoretical support for using illegitimate tasks as ego depletion triggering tasks in the dual-task paradigm.

Eventually, this research enriched the study of the boundary of illegitimate tasks. Regarding the boundaries of illegitimate tasks, existing studies mainly focused on individual characteristics or/and work context factors (Ma and Peng, 2019; Zhou et al., 2020). In this paper, this study attempted to explore the intervention conditions of illegitimate tasks from both work contextual factors and non-work contextual factors together. In terms of work context, there were differential findings in established studies regarding the moderating effects of coworker support (Fila and Eatough, 2020; Shin et al., 2020). This study focused on the emotional support aspect of coworker support, verifying the compensatory and spillover effects of coworker emotional support on the negative effects of illegitimate tasks and clarifying exactly what content form coworker support can take. In terms of non-work contexts, this research introduced leisure crafting as a moderating condition to explore the effects of non-work positive behaviors on work context and found that employees’ active leisure activities during non-work time moderated ego depletion brought about by illegitimate tasks, and further weakened the indirect negative effects of illegitimate tasks on work disengagement, expanding the boundary conditions study.

### Managerial Implications

On the basis of the findings of this study, the following management recommendations were proposed. Firstly, managers need to focus on optimizing task allocation decisions. Persons often detect the presence of illegitimate tasks in the work situation (Omansky et al., 2016; Thun, et al., 2018). In actual task scenarios, employees categorize certain specific tasks as illegitimate and develop work disengagement consequently, while managers may be unaware of it. Therefore, when managers conduct job design, they would better carry out some research interviews in advance to reasonably assess employees’ perceptions of tasks and determine the optimal work boundaries and assignment plan. On assigning tasks, managers are supposed to accurately communicate relevant information to ensure that task contents are implemented to specific members and refrain from blurred duty. During task execution, regular communication with employees should be included in the schedule to help them clarify job responsibilities and avoid the perception of illegitimate tasks.

Secondly, managers can adopt measures to curb the loss of resources of subordinates. The person who performs illegitimate tasks deserves to be compensated with some resources, such as material or spiritual rewards. These alternative incentives convey managers’ recognition about employees’ positive responses to illegitimate tasks and direct affirmation of employee worth, which can reduce their cognitive anxiety and negative emotions when facing illegitimate tasks. Moreover, managers can take targeted measures in recruitment and training to screen candidates with more self-control resources and further improve the relevant self-control ability through systematic training (Muraven and Baumeister, 2000).

Thirdly, formal or informal channels are used to guide and create a supportive and friendly workplace atmosphere. On the one hand, superiors can promote the functionalization of emotional support through formal channels. Specifically, this includes integrating mental health concerns into the framework of human resource management functions, setting up a special psychological care module for employees, so as to channel employees’ negative psychology in a timely and effective manner and prevent them from falling into a state of continuous depletion of psychological resources, thus cutting off the negative transmission path of illegitimate tasks. On the other hand, some informal channels that increase the emotional communication links among employees are supposed to encourage, such as “praise each other group” and other internal virtual communities to allow persons to interact with others and achieve the relief of negative psychology.

Fourthly, opportunities to engage in active leisure activities through benefits and other means can be provided for employees. Managers can incorporate benefits that fit the concept of “proactive recreation” into employee relationship management. Such benefits include the provision of infrastructure for leisure activities. Larger organizations can build their own sports complexes (e.g., badminton courts and swimming pools), while smaller organizations can partner with well-known or reputable sports organizations. In addition, managers can create an atmosphere that encourages employees to engage in leisure activities during their break time. For instance, by organizing informal club tournaments within departments or across the organization, the manager provides a platform for employees who excel in sports, arts, and other proactive leisure activities to express themselves and release stress.

### Limitations and Future Research

There were still some shortcomings in this study. Above all, although the article was data collected at two rounds, the contents were all filled out by a single source in a self-report style, making it difficult to avoid common method bias. Next, the scales used in this study were all established foreign scales and have good reliability and validity in this sample, but cultural differences made the translation of the scales inevitably biased, which affected employees’ understanding and responses and weakened scale credibility. Besides, in terms of control variables, this study only included the commonly used demographic variables in the analysis and failed to consider more variables that could potentially influence individual work disengagement, such as external pay incentives.

Meanwhile, scholars can consider the following directions for further research. Firstly, the impact of illegitimate tasks on positive employee working states, such as thriving at work, flow experiences, could be examined to explore the differential impact of illegitimate tasks on different types of working states. Secondly, it can be explored whether there is a positive effect of illegitimate tasks? Currently, illegitimate tasks exist as a hindering stressor, but do illegitimate tasks have the nature of challenging stressors in specific situations? Finally, scholars can investigate the boundary conditions of illegitimate tasks at the team and organization levels. There may be differences in the way employees respond to illegitimate tasks in different types of group atmosphere or organizational cultures. For example, in a collectivist culture, employees may comply with unreasonable task assignments from their leaders and be less likely to react negatively to illegitimate tasks.

## Conclusion

Illegitimate tasks have become a common stressor, and it is important to systematically recognize its negative effects and the inherent impact pathways, as well as to develop appropriate interventions. The findings of this paper have confirmed that there is a positive impact of illegitimate tasks on negative outcomes of employees (work disengagement), which is achieved through a progressive depletion of individual resources, and that this process involves employee cognitive load and negative emotions. To attenuate/eliminate the transmission of negative effects of illegitimate tasks, attempts could be made to supplement resources with coworker emotional support at work and self-compensation with leisure crafting at non-work.

## Data Availability Statement

The raw data supporting the conclusions of this article will be made available by the authors, without undue reservation.

## Author Contributions

SZ, YH, and ML involved in the study design and discussion. ML and SZ collected the data. SZ performed the data analysis and wrote the manuscript. YH developed the conceptual framework and revised the whole paper. ML reviewed it critically and gave important intellectual input. All authors contributed to the article and approved the submitted version.

## Funding

This work was supported by the National Natural Science Foundation of China (71972181), Program funded by the Humanities and Social Sciences Foundation of the Ministry of Education of China (19XJJC630001), and Fundamental Research Funds for the Central Universities, Zhongnan University of Economics and Law (202011005).

## Conflict of Interest

The authors declare that the research was conducted in the absence of any commercial or financial relationships that could be construed as a potential conflict of interest.

## Publisher’s Note

All claims expressed in this article are solely those of the authors and do not necessarily represent those of their affiliated organizations, or those of the publisher, the editors and the reviewers. Any product that may be evaluated in this article, or claim that may be made by its manufacturer, is not guaranteed or endorsed by the publisher.
